# New schedule of bevacizumab/paclitaxel as first‐line therapy for metastatic HER2‐negative breast cancer in a real‐life setting

**DOI:** 10.1002/cam4.803

**Published:** 2016-07-15

**Authors:** Katia Cannita, Stefania Paradisi, Valentina Cocciolone, Alberto Bafile, Lucia Rinaldi, Azzurra Irelli, Paola Lanfiuti Baldi, Luigi Zugaro, Rosa Manetta, Edoardo Alesse, Enrico Ricevuto, Corrado Ficorella

**Affiliations:** ^1^ Medical OncologyS. Salvatore Hospital, University of L'AquilaL'AquilaItaly; ^2^ Departement of Biotechnological and Applied Clinical SciencesUniversity of L'AquilaL'AquilaItaly; ^3^ Breast UnitS. Salvatore HospitalL'AquilaItaly; ^4^ Division of RadiologyS. Salvatore HospitalL'AquilaItaly; ^5^ Oncology Network ASL1 AbruzzoUOSD Oncology Territorial CareS. Salvatore Hospital, University of L'AquilaL'AquilaItaly

**Keywords:** Bevacizumab, metastatic breast cancer, new schedule, paclitaxel, real life

## Abstract

Angiogenesis plays an essential role in the growth and progression of breast cancer. This observational single center study evaluated the efficacy and safety of a new weekly schedule of bevacizumab/paclitaxel combination in the first‐line treatment of unselected, HER2‐negative, metastatic breast cancer (MBC) patients, in a real‐life setting. Thirty‐five patients (median age 56 years, range 40–81) with HER2‐negative MBC were treated with paclitaxel (70 mg/m^2^) dd 1,8,15 q21 (60 mg/m^2^ if ≥65 years or secondary Cumulative Illness Rating Scale) plus bevacizumab (10 mg/kg) every 2 weeks. Twenty‐two patients (63%) had ≥2 metastatic sites and 15 (43%) visceral disease. Eleven patients (31%) had a triple‐negative breast cancer (TNBC). A clinical complete response (cCR) was observed in 6 (17%) cases after a median of seven cycles, a partial response (PR) in 22 (63%), and a stable disease (SD) in 6 (17%) cases; the overall clinical benefit rate was 97%. In TNBC subgroup, cCR occurred in 1 (9%) case, PR in 8 (73%), and SD in 2 (18%). At a median follow‐up of 13 months (range 1–79 months), the median progression‐free survival was 11 months and the median overall survival was 36 months. No grade 4 adverse events occurred. The main grade 3 toxicities observed were neutropenia (11.4%), hypertension (5.7%), stomatitis (2.8%), diarrhea (2.8%), and vomiting (2.8%). The administration of weekly paclitaxel plus bevacizumab in this real‐life experience shows similar efficacy than previously reported schedules, with a comparable dose intensity and a good toxicity profile.

## Introduction

Angiogenesis is essential for the development of malignancies and plays a central role in the early stages of growth, invasion, and metastatic spread of breast cancer (BC), thus representing a reasonable therapeutic target. In vivo studies showed that the acquisition of a proangiogenic phenotype represents an early event in the development of BC 1. During mammary carcinogenesis, the expression of HIF‐1, a regulatory subunit of the hypoxia‐induced factor (HIF), increases proportionally to the progression from ductal hyperplastic lesion to ductal in situ carcinoma and invasive carcinoma 2, contextually to a growing expression of proangiogenic factors, including family members of vascular endothelial growth factors (VEGFs), fibroblast growth factors (FGF), transforming growth factor (TGF)‐B1, and thymidine phosphorylase (TP) 3. Unlike the other factors, VEGF is continuously expressed in all stages of carcinogenesis and it is the only angiogenic factor present throughout the entire tumor life cycle 4. This evidence is a solid biological rationale for the use of therapeutic agents able to interfere with the VEGF function. The recombinant monoclonal antibody bevacizumab is currently the most widely used and developed antiangiogenic drug in the treatment of BC, able to recognize all the isoforms of VEGF‐A, preventing its binding to the cellular receptor, and inhibiting the angiogenic and proliferative signal. In vivo studies showed that bevacizumab inhibits both proliferation and migration of endothelial cells induced by VEGF‐A; besides in some models of human BC, the treatment with bevacizumab was associated to a reduction in microvascular density 5. According to the Folkman model, bevacizumab would lead to the normalization of the tumor vasculature, resulting in a restoration of cancer cells susceptibility to chemotherapy. This theory is currently accompanied by the newer “action and reaction” model 6: bevacizumab enhances the antiangiogenic effect of chemotherapy, thus providing a rationale for the use of combination therapies. In particular, taxanes have an inhibitory action on the proliferation of endothelial progenitor cells, with an antiangiogenic effect at lower doses than those needed to inhibit the proliferation of cancer cells. The resulting hypoxia induces cancer cells to a kind of “reaction” through the autocrine production of proangiogenic agents, mainly belonging to the HIF‐1/VEGF pathway 7. Bevacizumab is able to inhibit this “reaction,” potentiating the antiangiogenic action of chemotherapy. This represents the biological assumption for the combination of paclitaxel with bevacizumab. Based on the preclinical evidence on the angiogenic activity of paclitaxel at lower doses, the E2100 study evaluated paclitaxel (90 mg/mq) on days 1, 8, 15 every 28 days in combination with bevacizumab (10 mg/kg) every 14 days as first‐line treatment for metastatic breast cancer (MBC) 8. Both E2100 and other two randomized phase III studies (AVADO and RIBBON‐1, which analyzed the impact of adding bevacizumab to chemotherapy in the first‐line treatment of HER2‐negative MBC), reported positive results in terms of overall response rate (ORR) and progression‐free survival (PFS). In the E2100 study, the addition of bevacizumab to paclitaxel doubled the PFS when compared with paclitaxel alone, without an overall survival (OS) gain 9, but a significant increase in grades 3–4 toxicities such as hypertension or proteinuria. The CALGB 9840 trial showed that weekly paclitaxel is superior to every‐3‐weeks paclitaxel in MBC, with a significant improvement in ORR and an important benefit in terms of PFS 10.

We report our real‐life experience with a new weekly schedule of paclitaxel in combination with bevacizumab (10 mg/kg every 14 days), which evaluated efficacy and safety of this first‐line regimen in unselected, HER2‐negative MBC patients.

## Patients and Methods

### Patients

From February 2009 to October 2014, 35 consecutive, unselected HER2‐negative MBC patients were treated with a modified schedule of bevacizumab plus paclitaxel at the Medical Oncology Department San Salvatore Hospital, University of L'Aquila. The study was performed according to the ethical rules for medical research involving human subjects, as stipulated by the WMA Declaration of Helsinki, and all patients gave their written informed consent.

Patients were treated in clinical practice, according to the indications of paclitaxel and bevacizumab approved by Agenzia Italiana del Farmaco (AIFA) for administration *in label* in Italian public hospitals, and published in the Gazzetta Ufficiale Repubblica Italiana (“Elenco dei Medicinali erogabili a totale carico del Servizio sanitario nazionale,” Gazzetta Ufficiale n. 146 del 24.06.08).

Women with MBC previously untreated with cytotoxic therapy were eligible for this study if they had histologically confirmed diagnosis of BC with HER2‐negative status, evaluated by immunohistochemistry (IHC) or fluorescence in situ hybridization (FISH) (HER2/neu oncoprotein expression negativity was assessed using the HerceptTest, scored 0 [absent] or 1+ [weak]; the negativity of the HER2/neu gene amplification was confirmed by FISH in case of HercepTest score 2+); World Health Organization (WHO) performance status ≤2; adequate hematological, renal, and hepatic function; life expectancy more than 3 months. Previous hormonal therapy for MBC or cytotoxic adjuvant chemotherapy was allowed. Exclusion criteria were pregnancy and breast feeding, uncontrolled severe diseases, cardiovascular diseases (uncontrolled hypertension, uncontrolled arrhythmia, ischemic cardiac disease in the last year), thromboembolic disease, coagulopathy, preexisting bleeding diatheses, proteinuria >1 g/24 h urine.

### Methods

The endpoints of the study were safety, ORR and clinical benefit rate (CBR, including responses and stability of disease for at least 6 months), PFS, and OS.

The clinical evaluation of safety, the clinical status, as well as liver and renal functions was assessed before each cycle; a complete blood count was obtained before each paclitaxel infusion. Urinalysis was performed before each bevacizumab infusion. Toxicity was registered weekly according to National Cancer Institute Common Toxicity Criteria (NCI‐CTC, version 3.0). Dose‐limiting toxicities (DLTs) requiring dose reduction or treatment delay/interruption were defined as grades 3–4 nonhematological toxicity (mainly represented by hypertension, neurotoxicity, hand–foot syndrome, diarrhea, proteinuria, mucositis, asthenia, epistaxis), grade 4 hematologic toxicity, febrile neutropenia, or any toxicity determining a >2 weeks treatment delay.

To better assess toxicity in the individual patient, we evaluated limiting TS (LTS) recently reported by our group 11, consisting of at least a DLT associated or not to other limiting or grade 2 toxicities. They were classified as LTS single site (LTS‐ss) if characterized only by a DLT; LTS multiple sites (LTS‐ms), if characterized by ≥2 DLTs or DLT associated to other, at least grade 2, nonlimiting toxicities.

Clinical evaluation of response was performed by CT scan every three cycles; PET or bone scan were added based on investigators’ discretion, according to the standard criteria of good clinical practice. The evaluation of response was performed according to the RECIST criteria 12.

PFS and OS were evaluated by Kaplan–Meier method 13. PFS was the time from the start of the treatment to disease progression or last visit; OS was the time from the start of the treatment to death or last visit.

### Treatment plan

**Figure 1:**
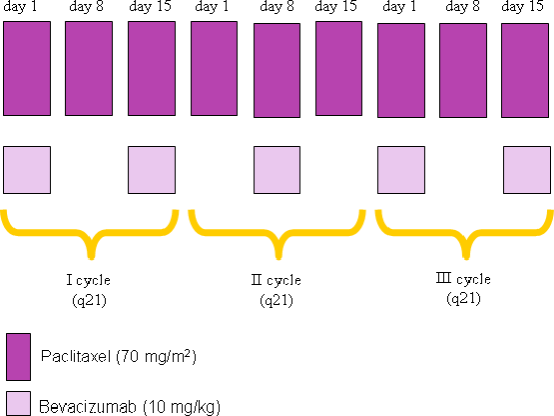
Treatment plan: paclitaxel 70 mg/m^2^ on days 1, 8, and 15 of a 21‐day cycle (weekly) and bevacizumab 10 mg/kg intravenously (i.v.) every 2 weeks; the paclitaxel dose for patients ≥65 years or with secondary Cumulative Illness Rating Scale (CIRS) was 60 mg/m^2^.

All patients received paclitaxel 70 mg/m^2^ on days 1, 8, and 15 of a 21‐day cycle (weekly) and bevacizumab 10 mg/kg intravenously (i.v.) every 2 weeks (Fig. 1); the paclitaxel dose for patients ≥65 years or with secondary Cumulative Illness Rating Scale (CIRS) was 60 mg/m^2^. The first bevacizumab dose was infused over 90 min; the subsequent infusions were reduced to 60 min for the second dose and to 30 min thereafter. Premedication was performed with i.v. dexametasone and chlorpheniramine maleate. Dose reduction of paclitaxel to 60 mg/m^2^ was planned in case of grade 3 or prolonged (>2 weeks treatment delay) grade 2 nonhematological toxicity or grade 4 hematological toxicity. Paclitaxel was permanently discontinued for severe hypersensitivity reactions or grade 4 nonhematological toxicity. Treatment was interrupted for proteinuria ≥2000 mg/24 h. Antihypertensive therapy was administered at the discretion of the investigator. Patients continued combined therapy until disease progression, unacceptable toxicity, or up to a maximum of 12 cycles.

Patients who discontinued paclitaxel without disease progression could continue bevacizumab monotherapy until disease progression or unacceptable toxicity with or without endocrine therapy.

### Statistical analysis

Survival curves were calculated by Kaplan–Meier method. PFS was calculated from starting of chemotherapy to disease progression or last visit. OS was the time from starting of chemotherapy to death or last visit 13.

## Results

Thirty‐five consecutive patients were enrolled. The median age was 56 years (range 40–81); 7 of the 35 (20%) aged ≥65 years. Infiltrating ductal carcinoma was the predominant histological subtype (80%), followed by the lobular subtype (14%). Hormonal receptors status was positive (ER positive and/or PgR positive) in 69% of cases, classified as Luminal A subtype in 17% (6 tumors) and Luminal B subtype in 52% (18 tumors), while triple‐negative tumors (TNBC) were 31%. Among all 35 treated patients, 6 (17%) had metastatic disease at diagnosis and 29 (83%) received first‐line treatment after tumor relapse, with a disease‐free interval >24 months in 12 of the 35 (34%) and ≤24 months in 17 of the 35 (49%) patients. Twenty‐seven patients (77%) had received adjuvant chemotherapy (anthracycline‐based, 30%; taxane‐based, 7%; anthracycline and taxane‐based, 44%; and other regimens, 19%), two patients (6%) had received neoadjuvant anthracycline and taxane‐based chemotherapy, 22 patients (63%) had received adjuvant endocrine therapy (tamoxifen, 41%; aromatase inhibitor, 41%; and tamoxifen and aromatase inhibitor, 18%). Median time from the end of adjuvant treatment to disease progression was 6 months (range 3–130 months). One patient had previously received endocrine therapy for MBC (fulvestrant). Sixty‐three percent of patients had two or more sites of metastasis and 43% of patients had visceral disease. WHO Performance Status was 0 in 29/35 patients (83%) and 1 in 6/35 patients (17%). Nine patients (26%) had a controlled hypertension just before starting chemotherapy (Table 1).

**Table 1:** Demographic characteristics of patients at baseline

	*N*. (%)
Median age (years)	56
Range	40–81
≥65 years	7 (20)
WHO performance status	
0	29 (83)
1	6 (17)
Medical history at study entry	
Hypertension	9 (26)
Tachycardia	–
Diabetes	1 (3)
Histological type	
Ductal	28 (80)
Lobular	5 (14)
Other	2 (6)
Hormonal receptor status	
ER‐positive and/or PgR‐positive	24 (69)
ER‐negative and PgR‐negative	11 (31)
Unknown	–
HER2 status	
Positive	–
Negative	35 (100)
Unknown	–
Prior treatment for primary BC	
Hormonal therapy	2 (6)
Chemotherapy (CT)	27 (77)
CT taxane	14 (40)
Disease‐free interval	
≤24 months	12 (34)
>24 months	17 (49)
Metastatic disease at diagnosis	6 (17)
N.MBC sites	
1	13 (37)
≥2	22 (63)
Location of disease	
Visceral	15 (43)
Nonvisceral	20 (57)
Bone only	8/20 (40)
Metastatic sites	
Bone	23 (66)
Liver	7 (20)
Lung	9 (26)
Other	20 (57)

BC, breast cancer; WHO, World Health Organization.

Overall, 247 treatment cycles were administered; the median number of cycles was 6 (range 3–12).

### Safety

As far as safety is concerned, in our study no grade 4 toxicities were observed, with the exception of alopecia. The grade 3 toxic effects were nonfebrile neutropenia (4/35, 11.4%), hypertension (2/35, 5.7%), oral mucositis (1/35, 2.8%), fatigue (1/35, 2.8%), diarrhea (1/35, 2.8%), and vomiting (1/35; 2.8%). Among the most significant grade 2 side effects, hypertension was observed in 40% (14/35) of patients, well managed by medical treatment; neuropathy in 11.4% (4/35), ≥2 weeks in just one patient; diarrhea in 8.5% (3/35) patients; proteinuria ≥2 weeks in 2.8% (1/35) and onychodystrophy in 2.8% (1/35) patients (Table 2). The most relevant toxicity in the subgroup of elderly patients (*n* = 7, ≥65 years) was grade 2 hypertension (6/7, 85.7%; in three cases a controlled hypertension was already presents at baseline); the seventh patient experienced grade 1 hypertension. In this subgroup of patients, grade 3 adverse events (AEs) were represented by diarrhea (1/7, 14%) and vomiting (1/7, 14%). Grade 2 AEs were fatigue (1/7, 14%), diarrhea (1/7, 14%), rhinitis (1/7, 14%), neuropathy (1/7, 14%), and onychodystrophy (1/7, 14%). Grade 1 AEs were nausea (2/7, 28%), epistaxis (1/7, 14%), fatigue (1/7, 14%), diarrhea (1/7, 14%), rhinitis (1/7, 14%), myalgia (1/7, 14%), neuropathy (1/7, 14%), stomatitis (1/7, 14%), and constipation (1/7, 14%).

**Table 2:** Toxicity (all grades)

*Grade*	Patients			
	35			
	1	2	3	4
Neutropenia (*n*, %)	8 (22.8)	5 (14.2)	4 (11.4)	–
Anemia (*n*, %)	3 (8.5)	–	–	–
Hypertension (*n*. %)	–	14 (40)	2 (5.7)	–
Epistaxis (*n*, %)	27 (77.1)	2 (5.7)	–	–
Proteinuria (*n*, %)	–	1 (2.8)	–	–
Stomatitis (*n*, %)	15 (42.8)	2 (5.7)	1 (2.8)	–
Gingivitis (*n*, %)	1 (2.8)	1 (2.8)	–	–
Rhinitis (*n*, %)	3 (8.5)	1 (2.8)	–	–
Conjunctivitis (*n*, %)	3 (8.5)	–	–	–
Fatigue (*n*, %)	26 (74.2)	2 (5.7)	1 (2.8)	–
Constipation (*n*, %)	6 (17)	–	–	–
Diarrhea (*n*, %)	8 (22.8)	3 (8.5)	1 (2.8)	–
Nausea (*n*, %)	4 (11.4)	–	–	–
Vomiting (*n*, %)	–	–	1 (2.8)	–
Neuropathy (*n*, %)	14 (40)	4 (11.4)	–	–
Headache (*n*, %)	5 (14.2)	–	–	–
Myalgia (*n*, %)	18 (51.4)	1 (2.8)	–	–
Allergic reactions (*n*, %)	1 (2.8)	–	–	–
Onychodystrophy (*n*, %)	3 (8.5)	1 (2.8)	–	–
↑ Transaminase (*n*, %)	5 (14.2)	1 (2.8)	–	
Alopecia (n, %)	–	–	–	35 (100)

Five patients reported LTS, all represented by LTS‐ms, characterized by G3 hypertension plus G2 proteinuria lasting ≥2 weeks plus G3 neutropenia in one patient; G3 hypertension plus G3 fatigue plus G2 neuropathy in one patient; G3 mucositis plus G2 hypertension in one patient; G3 diarrhea plus G3 vomiting plus G2 neuropathy plus G2 hypertension in one patient; and G2 neuropathy lasting ≥2 weeks plus G2 hypertension plus G2 myalgia plus G2 diarrhea in one patient.

### Response and survival analysis

At the time of data collection, five patients were still on treatment.

A clinical complete response (cCR) was achieved in 17% (6/35) of patients, a partial response (PR) in 63% (22/35) and stable disease (SD) in 17% (6/35) of patients. The ORR (cCR + PR) was 80% and the CBR (cCR + PR + SD) was 97%. Most patients (66% [23/35] of all patients; 82% [23/28] of patients achieving objective response) had an objective response after a median of three cycles of chemotherapy.

In the subgroup of TNBC, one patient (9%) achieved a cCR, eight patients (73%) had a PR, and two patients (18%) a SD, for an ORR of 82% and a CBR of 100%.

In the subgroup of elderly patients, treated with a paclitaxel dose of 60 mg/m^2^, six of the seven patients (86%) reported a PR and one patient (14%) had a SD.

**Figure 2:**
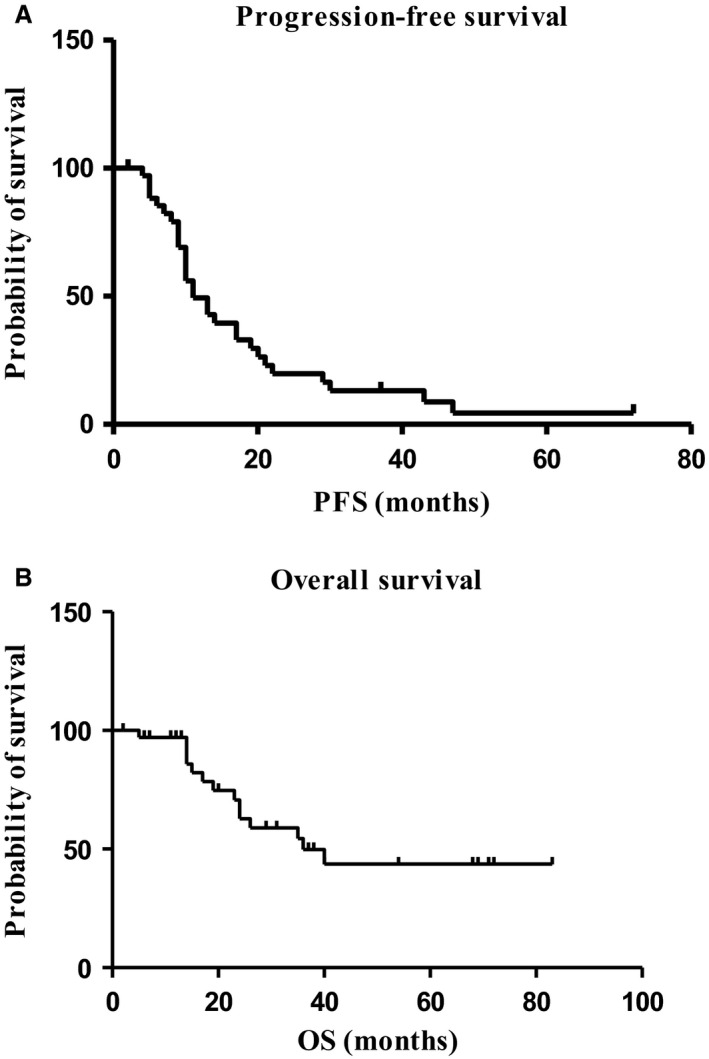
Kaplan–Meier curves of disease‐free survival (DFS) (A) and overall survival (OS) (B). After a median follow‐up of 13 months (range 1–79 months), the median PFS was 11 months and the median OS 36 months. PFS, progression‐free survival.

After a median follow‐up of 13 months (range 1–79 months), the median PFS was 11 months and the median OS 36 months (Table 3). Figure 2 shows Kaplan–Meier curves related to PFS and OS.

**Table 3:** Activity and efficacy

	Clinical response(35 patients)	
	*n*.	%
Complete response (CR)	6	17
Partial response (PR)	22	63
Stable disease (SD)	6	17
Progression disease	1	3
Clinical benefit rate (CR + PR + SD)	31	97
Median duration of follow‐up	13 months	
Progression‐free survival	11 months	
Overall survival	36 months	

## Discussion

This is a small real‐life observational study evaluating a modified schedule of paclitaxel and bevacizumab combination regimen as first‐line treatment of HER2‐negative MBC patients.

The choice of adopting the weekly schedule without the “intermittent week” stems from the following considerations. In BC chemotherapy, the weekly administration of paclitaxel, as demonstrated by several studies, is more effective than the thrice weekly schedule 10. The activity of paclitaxel is directly related to the cell cycle and a more frequent administration enhances its proapoptotic and antiangiogenetic properties, thus increasing the antineoplastic effect 14, 15, 16.

On the other hand, from a tolerability point of view, the weekly schedule of paclitaxel (80 mg/m^2^) significantly increases neurotoxicity compared to the thrice weekly schedule, as shown by the phase III CALGB 9840 trial 10, a consideration leading to the choice of an “intermittent weekly” schedule in the E2100 study 9. Miller et al. reported limiting grades 3–4 toxicity represented by sensory neuropathy in 23.6% of patients, hypertension in 14.5%, infection in 9.3%, and fatigue in 8.5%. In clinical practice, these well‐known side effects, even if low grade, can lead to a reduced compliance to treatment and often affect quality of life.

Thus, considering that traditional chemotherapy and anti‐VEGF agents are synergistic in their action, as angiogenesis inhibitors sensitize to chemotherapy, it is rational to hypothesize that the combination requires lower doses of chemotherapy 17.

In this regard, in E2100 reductions of paclitaxel due to paclitaxel‐related toxicities occurred more frequently in patients in the bevacizumab/paclitaxel group, thus preserving paclitaxel effect with bevacizumab sensitization. As previously mentioned, on the one hand taxanes affect vascular endothelium and synergize with bevacizumab at picomolar doses to prevent vascular sprouting 18 and, on the other hand, bevacizumab can modify the delivery of chemotherapeutic agents to tumors by modifying tumor vascularity 19, this synergism could have decreased the toxicities while maintaining antitumor efficacy with reduced doses of chemotherapy 17.

In order to improve the tolerability of bevacizumab and paclitaxel combination, in our real‐life experience we used a weekly paclitaxel dosage of 70 mg/m^2^. Even if lower than 90 mg/m^2^ used in the pivotal E2100 trial, this dose determines a highest weekly dose intensity (DI) (70 vs. 67.5 mg/m^2^), due to the absence of the “intermittent week.” In our study, the dose of paclitaxel, but not of bevacizumab, was reduced by 10% also in patients with secondary CIRS. With such dose and schedule modulation of paclitaxel, associated with biweekly bevacizumab 10 mg/kg, no grade 3 neuropathy events were observed and grade 2 neurotoxicity occurred only in 11.4% of patients, with one case of limiting (lasting more than 2 weeks) sensory neuropathy. Even if this analysis has been performed in a limited population, the incidence of limiting neuropathy was significantly lower than that reported in the E2100 trial, where grades 3–4 neuropathy occurred in 23.6% of patients in the bevacizumab/paclitaxel arm versus 17.6% in the paclitaxel‐alone arm. It was suggested that this difference could be related to a prolonged paclitaxel exposure rather than to bevacizumab exposure, because in the AVADO trial grade ≥3 neuropathy occurred in 3–4% of patients receiving bevacizumab versus 2% with placebo 20. Moreover, no grade 4 toxicities were observed, with the exception of alopecia. In our real‐life experience, the rate of grade 3 hypertension was considerably lower than that observed in E2100 study (5.7% vs. 14.5%) 9 and comparable to that of ATHENA study (4.4%), which evaluated bevacizumab 10 mg/kg every 2 weeks in combination with a taxane‐based chemotherapy in the routine oncology practice 21. In elderly population (≥ 65 years), no grade 3, but only grade 2 hypertension events were observed, with a very high incidence (85.7%). The higher incidence of hypertension in older patients confirmed the results of the subanalysis of the ATHENA trial on patients ≥70 years. In this report, only hypertension and proteinuria were more common in older than in younger patients (grade ≥3 hypertension: 6.9% vs. 4.2%, respectively; grade ≥3 proteinuria: 4.0% vs. 1.5%, respectively). Moreover, hypertension was more common in patients with active hypertension at baseline than in those with normal blood pressure (48% vs. 37%, respectively, in patients ≥70 years; 38% vs. 26%, respectively, in patients <70 years) 21.

In our study, two patients (5.7%) required bevacizumab withdrawal and four patients (11.4%) a paclitaxel dose reduction. Overall, five patients (14.2%) required an adjustment/interruption of treatment, as one patient both discontinued bevacizumab and reduced paclitaxel. These results show that this regimen is well tolerated with a low incidence of grades 3–4 side effects. We observed a very high adherence to treatment, with 247 administered cycles and a median of six cycles (range 3–12) delivered per patient.

The efficacy of bevacizumab plus paclitaxel treatment observed in our real‐life study was very encouraging: the ORR was 80% (cCR 17%; PR 63%), higher than data reported in the phase III trials evaluating the approved bevacizumab dose combined with a taxane (50% in E2100 study, 64% in AVADO study, 51% in the taxane/anthracycline cohort of RIBBON‐1 study) and 52% obtained in the ATHENA trial 9, 20, 22, 23. Moreover, the CBR was 97% and 66% of all patients obtaining a PR after a median of three cycles of chemotherapy. Probably, the high compliance to chemotherapy, together with the good safety profile and the maintenance of an adequate quality of life, allowed us to obtain very encouraging results also in a “frail” population, like patients with secondary CIRS.

The ORR and CBR achieved in the subgroup of TNBC patients, 82% and 100%, respectively, confirms conclusions of subgroups analyses of the ATHENA and E2100, AVADO, and RIBBON trials, showing that first‐line chemotherapy in combination with bevacizumab is an active regimen in poor‐prognosis patients with limited treatment options, like patients with metastatic TNBC 24, 25. In this regard, the GeparQuinto trial has suggested that, also in the neoadjuvant setting, patients with TNBC benefit more from adding bevacizumab to anthracycline/taxane‐based chemotherapy than non‐TNBC patients, being the pathological complete response rate 39.3% and 27.9% (*P* = 0.003) with and without bevacizumab, respectively, for TNBC patients and 7.7% and 7.8%, respectively, for patients with hormone receptor‐positive tumors (*P *= 1.00) 26.

After a median follow‐up of 13 months (range 1–79 months), the median PFS was 11 months and the median OS 36 months. The median PFS is similar to that of the previously mentioned pivotal studies (11.3, 10, 9.2, and 9.5 months in E2100, AVADO, taxane/anthracycline cohort of RIBBON‐1, and ATHENA study, respectively) 9, 20, 22, 23, while the median OS is slightly higher (26.7, 30.2, 27.5, and 25.2 months in E2100, AVADO, taxane/anthracycline cohort of RIBBON‐1, and ATHENA study, respectively) 9, 20, 22, 23, probably due to the additional lines of systemic treatment that patients received after first‐line chemotherapy.

## Conclusions

This modified schedule of bevacizumab and paclitaxel combination regimen used in a real‐life clinical setting results in a very high tolerability, with efficacy results consistent with those reported in other clinical trials on the first‐line treatment of HER2‐negative MBC patients.

Biomarkers able to predict the response to antiangiogenic therapies are urgently needed, as they could improve patient selection and drive clinicians’ choices. A biomarker analysis on this study population would help clarifying which patients could benefit more from administered drugs.

## Conflict of Interest

The authors declare that they have no conflict of interest.
